# “It’s a Battle… You Want to Do It, but How Will You Get It Done?”: Teachers’ and Principals’ Perceptions of Implementing Additional Physical activity in School for Academic Performance

**DOI:** 10.3390/ijerph14101160

**Published:** 2017-09-30

**Authors:** Vera van den Berg, Rosanne Salimi, Renate H. M. de Groot, Jelle Jolles, Mai J. M. Chinapaw, Amika S. Singh

**Affiliations:** 1 Department of Public and Occupational Health, the Amsterdam Public Health Research Institute, VU University Medical Center, 1081 HV Amsterdam, The Netherlands; v.vandenberg@vumc.nl (V.v.d.B.); rosannesalimi@hotmail.com (R.S.); m.chinapaw@vumc.nl (M.J.M.C.); 2 Welten Institute—Research Centre for Learning, Teaching and Technology, Open University of the Netherlands, 6419 AT Heerlen, The Netherlands; renate.degroot@ou.nl; 3 Department of Complex Genetics, School for Nutrition, Toxicology and Metabolism/Faculty of Health, Medicine and Life Sciences, Maastricht University, 6211 LK Maastricht, The Netherlands; 4 Centre for Brain and Learning, Faculty of Psychology and Education, LEARN! Institute, VU University Amsterdam, 1081 HV Amsterdam, The Netherlands; j.jolles@vu.nl

**Keywords:** physical activity, academic performance, school setting, feasibility, perceptions teachers, perceptions principals, intervention development, interviews, qualitative research

## Abstract

School is an ideal setting to promote and increase physical activity (PA) in children. However, implementation of school-based PA programmes seems difficult, in particular due to schools’ focus on academic performance and a lack of involvement of school staff in program development. The potential cognitive and academic benefits of PA might increase chances of successful implementation. Therefore, the aim of this qualitative study was: (1) to explore the perceptions of teachers and principals with regard to implementation of additional PA aimed at improving cognitive and academic performance, and (2) to identify characteristics of PA programmes that according to them are feasible in daily school practice. Twenty-six face-to-face semi-structured interviews were conducted with primary school teachers (grades 5 and 6) and principals in The Netherlands, and analysed using inductive content analysis. Teachers and principals expressed their willingness to implement additional PA if it benefits learning. Time constraints appeared to be a major barrier, and strongly influenced participants’ perceptions of feasible PA programmes. Teachers and principals emphasised that additional PA needs to be short, executed in the classroom, and provided in “ready-to-use” materials, i.e., that require no or little preparation time (e.g., a movie clip). Future research is needed to strengthen the evidence on the effects of PA for academic purposes, and should examine the forms of PA that are both effective as well as feasible in the school setting.

## 1. Introduction

Schools are considered an ideal setting for the promotion of a healthy life style among children, such as regular physical activity (PA) [1]. It is well-known that regular participation in PA is beneficial for children’s physical and mental health [2,3]. Recent studies also suggest that participation in PA can result in immediate as well as longer-term improvements of children’s cognitive and academic performance [4,5]. Many school-based programmes have been developed with the aim to increase PA levels of children and adolescents [6]. However, adequate implementation of PA programmes in schools has been shown to be very difficult, resulting in low adherence rates and little or no effects on increasing children’s PA levels [6,7].

Studies on the effectiveness of PA interventions are increasingly accompanied by process evaluation studies to gain insight into the feasibility of the intervention, and barriers and facilitators of the implementation process [8]. A recent comprehensive review concluded that a lack of time is the most prevalent barrier for school staff hindering the implementation of school-based PA interventions [6]. Due to high pressures of academic performance and reaching educational targets as formulated by the government, schools mostly focus on teaching academic core subjects such as mathematics, language, and reading [9,10]. Consequently, activities that are not directly related to these subjects, such as PA, are often eliminated [11,12]. Other important barriers include an unsupportive school climate (e.g., lack of principal support and monitoring), and low teachers’ motivation and readiness to change [1,6].

Although process evaluation studies provide valuable information to adapt interventions developed by health professionals, school professionals are rarely involved in the development of school-based PA programmes [1,8,12]. As a result, many programmes do not align with school professionals’ needs, and appear to be unfeasible in daily school practice [12,13]. Consequently, programmes are often not implemented as intended, which complicates the interpretation of the effectiveness of these programmes [6,7]. Therefore, it is important to involve teachers and principals before or during early intervention development [1,8,12,14,15]. Besides, there is a need for input from a variety of teachers and principals, including those who are least motivated and not yet involved in implementation of PA programmes [1,15]. While teachers are designated to deliver PA programmes, principals have a significant role in supporting the implementation, as they are involved in establishment of school policy and school-wide supervision [12,16]. Therefore, both groups play an important role when it comes to the development of school-based PA interventions.

In particular, we lack knowledge on teachers’ and principals’ perceptions of feasible PA programmes in schools with regard to how often, how long, and in which setting (e.g., classroom, playground, physical education, recess) PA can be implemented [1]. Some previous process evaluation studies reported that teachers prefer classroom-based PA that is enjoyable for students, short in duration and easy to manage, with some teachers preferring PA related to academic content [14,17,18]. More insight is also needed in what, according to teachers, are the most appropriate types of PA for students in the highest grades of primary school [14].

Previous school-based PA interventions have mainly focused on promotion of children’s health; however, several studies reported that not all teachers and principals consider health promotion their responsibility [14,16,19]. A recent review reported small, but positive effects of PA on several cognitive and academic outcomes of children and adolescents [20], which may better align with teachers and principals’ needs. To date, however, little is known about the perceptions of teachers and principals to implement PA aimed at enhancing cognitive and academic performance rather than health. Only few studies explored the perceptions of school staff with regard to implementation of PA interventions with the purpose to improve academic performance, or to improve both health and academic outcomes [14,15,21,22]. The majority of teachers in these studies was positive about the use of the PA programmes, since they experienced learning benefits for their students [14,15,21]. In a study of Gately and colleagues, teachers perceived the use of the PA programme as a loss of time [22]. The generalisability of these studies is limited due to a small number of participating schools and respondents. Besides, each study evaluated the use of one specific PA intervention, e.g., 60 min additional physical activity per day [21], movement integration in academic subjects [14,15,22] or PA sessions in the classroom [14]. To our knowledge, there are currently no published results of studies that focus on how teachers and principals, in general, perceive the use of PA in school to improve children’s cognitive and academic performance. Therefore, in the current qualitative study we (1) explored the perceptions of teachers and principals with regard to implementation of additional PA in school aimed at improvement of cognitive and academic performance of 10 to 13 years old students in grades 5 and 6 of primary education in The Netherlands; and (2) identified characteristics of PA programmes that, according to school staff, are feasible and sustainable in daily school practice.

## 2. Materials and Methods

### 2.1. Study Design

The current study has a qualitative research design with an inductive approach; we identified themes from the data, which allowed us to gather information from teachers and principals’ perspectives [23]. We conducted semi-structured interviews, which are widely used in qualitative research and form an appropriate method to provide in-depth knowledge on the views, experiences, opinions and beliefs of participants [24].

### 2.2. Recruitment of Participants and Ethics

A purposive sampling method was used to recruit participants [25]. Selection criteria included: (1) schools in urban, suburban and rural areas in different regions across The Netherlands; (2) classroom teachers in grades 5 and/or 6 in primary education, or school principals in primary education, and (3) schools that currently do not implement school-wide PA interventions in addition to their regular physical education classes.

Teachers and principals of 53 regular primary schools were approached by email and follow-up phone calls to participate. We emphasized in our recruitment email that teachers and principals who are enthusiastic as well as those who are sceptical on the subject “PA and academic performance” were welcome to participate in our study. Twenty schools (38%) did not respond. Nineteen schools (36%) were not able to participate due to busy school schedules (n = 8), an overwhelming number of requests to participate in research projects (n = 3), already participating in other research projects (n = 1), no interest in the research topic (n = 1), or without specific reason (n = 6). Fourteen schools (26%) agreed to participate in the study with either their principal(s), their teacher(s), or both.

All participants received written information of the purpose and procedure of the study and signed informed consent prior to participation. The Medical Ethical Committee of the VU University Medical Center Amsterdam concluded that the study does not fall within the scope of the Medical Research Involving Human Subjects Act, and approved the study protocol (2014.363).

### 2.3. Interview Guide

Two interview guides (teacher and principal version) were developed following the recommendations of Creswell [25]. The interview started with a number of short questions regarding background variables of the participants, followed by two opening questions aimed at introducing the subject and building a working relationship with the interviewer. Thereafter, the core questions were addressed (see Appendix A). Probes and follow-up questions were used to encourage participants to talk, provide examples and elaborate on their ideas and opinions [24].

We developed a protocol and organized a training day to make sure that the interview guides were applied equally by our four interviewers. The interview guides were pilot tested in two preliminary interviews and evaluated with the interviewers. Based on the results of the pilot testing, the order of the questions was slightly adapted.

### 2.4. Data Collection

The four trained interviewers conducted 26 semi-structured interviews in Dutch concurrently between April and July 2015. At each school, one or two principals and/or one to four teachers were interviewed individually. The interviewers visited participants at their school, where the interviews were held in a quiet, private setting. Before the start of the interview, information on the aim of the study, the duration of the interview, and anonymity and confidentiality was provided. The importance of participants’ own opinions, experiences and ideas was emphasized.

All interviews were audio- or video-recorded and transcribed afterwards by two interviewers and Vera van den Berg. The duration of the interviews ranged from 9 to 35 min (mean 20 min) for teachers and 17 to 26 min (mean 19 min) for principals. The raw data consisted of 70 transcript pages from the principal interviews and 90 pages from the teacher interviews (Times New Roman, size 12, single spaced).

### 2.5. Data Analysis

**Figure 1:**
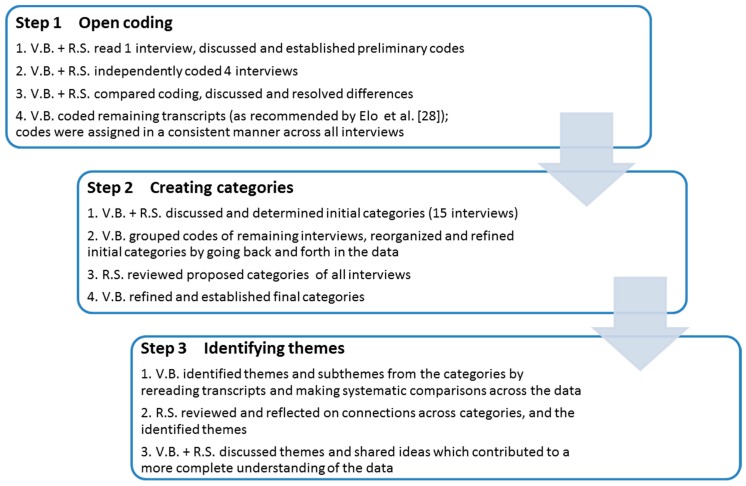
Overview of the data analysis process. Note: V.B.: Vera van den Berg; R.S.: Rosanne Salimi.

The data analysis started after the data collection. Inductive qualitative content analysis was used to systematically identify and extract categories and themes from the data [26,27]. Three core steps of qualitative content analysis were followed: (1) selecting the unit of analysis and open coding; (2) creating categories; and (3) establishing themes [26,28]. The analysis consisted of an iterative process, with all data processed in Microsoft Word and Excel by Vera van den Berg and Rosanne Salimi. The data of the teacher and principal interviews were analysed separately. Figure 1 shows an overview of the data analysis process.

In step 1 transcripts were read repeatedly, relevant text fragments were selected, and preliminary codes were allocated. Codes were supported by short phrases of the content of the text fragments, and coded transcripts were checked and revised repeatedly in order to make sure that identified codes were corroborated across the interviews [28]. A short summary of each interview was written. In step 2, categories were created by grouping text fragments with similar codes. During this phase, categories were reorganized and refined until final categories were established [26]. In step 3, themes and subthemes were identified by grouping related categories. Interview transcripts were reread, systematic comparisons were made across the data, and overviews were created to reflect on the themes arising from the interviews.

For example, categories “small classroom size”, “many students in the classroom”, and “full schedules of the external sports hall”, were grouped together under the subtheme “space constraints”, and theme “barriers”.

## 3. Results

### 3.1. Participants

In total, 15 fifth and sixth-grade teachers and 11 principals of the 14 schools participated. Schools were located in urban (n = 8), suburban (n = 2), and rural (n = 4) areas across The Netherlands. Teachers had a mean age of 41 years (range 25–61); principals were on average 48 years old (range 34–59). Work experience ranged from 1–37 years (mean 13.4) and 1–30 years (mean 11) in teachers and principals, respectively. Of the participants, 80% were female.

### 3.2. Interviews

Although data saturation was reached after analysis of approximately three quarter of the interviews, we analysed all interviews. One interview with a principal was excluded, since the interviewer used the teacher version instead of the principal version of the interview guide.

**Figure 2:**
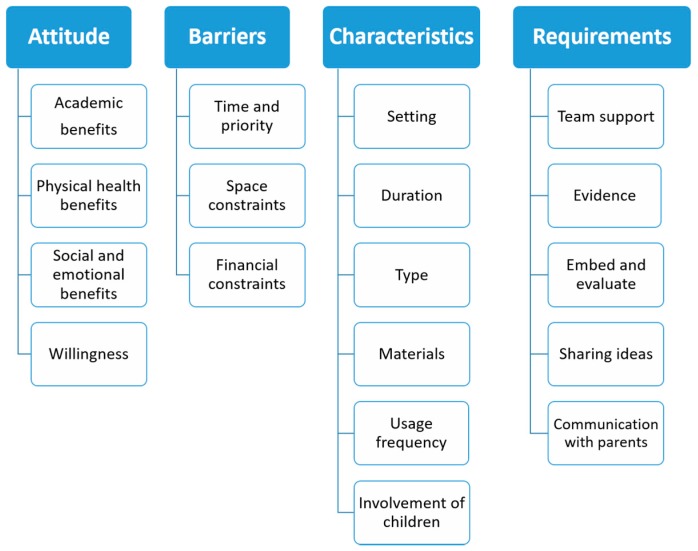
Themes and subthemes resulting from the data analysis.

The results of the interviews were organized around four key themes: attitude towards PA, barriers towards additional PA, characteristics of feasible PA programmes, and requirements for implementation of PA programmes in school. The findings were supported by quotations from the interviews, translated from Dutch into English. Figure 2 shows an overview of the themes and subthemes.

#### 3.2.1. Attitude towards PA

All teachers and principals showed a positive attitude towards PA, and gave several reasons why they believed PA is important for their students.

*Academic benefits*. The most common belief of all participants was that PA positively influences children’s ability to learn. Teachers indicated from experience that the majority of children need to move in order to release their energy and to recover attention. For example, teacher #10 noticed “Even if you just do a little bit of exercise, because a lot of children really need it, after that you can start up your attention span again”.

*Physical health benefits*. Almost all teachers and principals mentioned the importance of PA for physical health, and some (four teachers and seven principals) believed that “if you’ve got a healthy body, you perform better cognitively” (teacher #3). However, some participants argued that their “core business is getting children to learn” (principal #7).

*Social and emotional benefits*. A group of five teachers and five principals also observed the benefits of PA for children’s social skills and self-confidence. “They learn to function in a group, to collaborate, learn to deal with losing… But also to be in the limelight if you happen to be good at basketball and maybe can’t learn very easily, you notice that a child gets a kick out of it” (principal #4).

*Willingness*. Overall, teachers and principals were positive towards implementation of additional PA in school. However, they mentioned that their willingness depended on the perceived needs of children to move and the efficiency for learning. Teacher #7 mentioned for example: “If it isn’t showing any results, I say stop doing it and leave them sitting”. In addition, the willingness was restricted to certain forms of PA (see Section 3.2.3). Both groups discussed that willingness may vary across colleagues, because “some people don’t see the use of it very much” (teacher #1).

#### 3.2.2. Barriers towards Additional PA

Nearly all teachers and principals indicated a gap between their intention and actual implementation of additional PA, which they related to time constraints (major barrier), lack of financial support, and space constraints.

*Time and priority*. All teachers and principals emphasized a lack of time as a major barrier hindering implementation of PA programmes, and this topic recurred repeatedly throughout all interviews. The perceived lack of time was related to high demands of the government and parents on academic performance, competing priorities, and full school schedules. Teachers expressed that they feel overwhelmed by several responsibilities on top of teaching, like administrative tasks. Overall, it appeared that, according to teachers and principals, implementation of additional PA currently has no priority in school.

“Sometimes you’re trapped between the everyday rush, inspection reports, entrance exams, and the things that have to be done… The question is, whether that’s possible, and whether it’s desirable in our system, our school system in The Netherlands… because you’re judged on how good your arithmetic and spelling results are” (teacher #5).

*Space constraints*. According to the principals, implementation of additional PA in form of additional physical education classes is hindered by the available space. Principal #5 mentioned for example: “Then you have to go to the gym or the sports hall. And you have to take the timetable into account, because other schools also use the same facilities”. Concerns with regard to limited space in the classroom for PA were reported by one third of the teachers: “Make allowances for the fact that the space available—you can see that here—is often limited” (teacher #15).

*Financial constraints*. A lack of financial support from the government was mentioned by more than half of the principals and a small group of teachers. This barrier was related to hiring a physical education teacher or professional education of classroom teachers, and playground or sport facilities.

“We won’t be getting an extra PE teacher, which is a real shame. It would be great, but you need more money from the government. It would be really good if you had money to redesign your playground more effectively” (principal #10).

#### 3.2.3. Characteristics of Feasible PA Programmes

Teachers and principals’ ideas of feasible forms of PA followed logically from the perceived barriers. The vast majority of participants emphasized that additional physical education classes are not feasible in school. In general, teachers provided more concrete ideas than principals.

*Setting*. In terms of feasibility and relative to the time barrier, teachers reported that PA programmes need to be implemented by teachers themselves in their classroom. Teacher #2 mentioned: “If you have to go to another location all the time, it just won’t work. You’re stuck with the time factor... If I start doing things here in the corridor, the other classes won’t be very happy”. In addition, being active with the whole class was considered important for supervision and safety reasons. “If they start moving around in small groups for example, someone has to be around, otherwise it’s irresponsible” (teacher #7).

*Duration*. All teachers and principals agreed that additional PA needs to be of short duration. Teacher #4 said for example: “You can find 5 or 10 min somewhere during the school day”. According to both groups, a feasible duration varies between 1 and 5, up to a maximum of 10 min.

*Type*. Most teachers (n = 13) and principals (n = 7) considered PA breaks within or between academic lessons feasible. Several teachers suggested to use dance, movement clips, movement games or exercises around the table, such as jumping and running in place. Some teachers mentioned that there are short movement games available, with which they already had positive experiences.

Another suggested type of PA was integration of PA with academic subjects (5 teachers and 5 principals). A recurrent example was exercising while performing math sums. Teacher #2 explained: “That’s a way of calculating that focuses on automation, it’s about movement, so every time you do something, you jump”. However, some participants thought it would be difficult to use such methods in the highest grades of primary education (10–12 year olds). A small group of two teachers and four principals also mentioned other forms of being active while learning. For example, games with alternately sitting and standing, or walking to corners of the classroom when answering questions.

Furthermore, some teachers (n = 4) and the majority of principals (n = 7) saw opportunities to increase PA levels outside learning hours. They discussed the possibility of stimulating PA during recess, since not all children are physically active during recess time. They suggested to let children choose from activities, or by giving them cards with PA exercises, and providing more playground facilities. Also, PA interventions after school hours were mentioned by six principals. “I also know that there are out-of-school situations where sports instructors are hired to get children to exercise. It would be very interesting to see if we could make it accessible for all children… to create a kind of extended school day” (principal #1).

*Materials*. Almost all teachers and principals reported that it is important that PA interventions “don’t cost too much time to prepare” (teacher #9). They recommended materials that are ready to use, for example on the digital screen, in a booklet, or in a card box.

*Usage frequency*. The majority of teachers (n = 11) reported that they would implement PA spontaneously/incidentally, when children lost their attention, while a small group (i.e., 3 teachers) preferred structural implementation.

*Involvement of children*. A quarter of all teachers and several principals pointed out the importance of involving the children when designing PA programmes. They emphasized that PA must be varied, fun, challenging, and appropriate for the age group, which could best be explained by the children themselves. Several teachers reported that “up until the second last year at school (grade 5), things are just fine, it’s easy to make it fun… but in the final year (grade 6), they think it’s more important that it’s cool” (teacher #15).

#### 3.2.4. Requirements for Implementation of Additional PA in School

Teachers and principals addressed several prerequisites for successful implementation and sustainability of PA programmes in school.

*Team support*. Everyone emphasized that school-wide team support is essential when trying to implement additional PA in school. Teacher #5 said for example: “We’re fantastic at imagining all kinds of complications ... which is why I say you have to make a statement as a team that you want this. That’s the most important thing”. And after the decision has been made “it’s important to make clear agreements with each other” (principal #6).

*Evidence*. To establish team support, both groups reported the importance of a clear rationale, supported by evidence about the usefulness of PA for cognition/academic performance. Participants emphasized that providing this knowledge is especially important to gain support from colleagues who are less familiar or willing to use PA. In this respect, principal #1 said: “If a teacher is not convinced it’s going to help and that it’s practical, it doesn’t matter how many programmes you set up, nothing much will change. Even if you say it’s been agreed within the school and we’re going to do it”.

*Embed and evaluate*. Teachers and principals discussed the importance of structurally incorporating PA programmes in the school schedule, such that teachers are aware that they need to implement it. For example, teacher #11 mentioned: “Regular set periods during the week would be great. That you think to yourself, oh yeah, that’s still to come”. In addition, several principals reported that PA programmes must be part of the school policy in order to successfully integrate it in school.

Both teachers and principals discussed the importance of regular evaluation within the team to prevent relapse. The majority recommended an internal PA-commission or coordinator responsible for the implementation. Teacher #8 explained: “You can say that everyone takes care of it themselves, but they coordinate it, or propose ideas, or monitor it. Because we all have so much to do that if nobody specifically reminds you, it just doesn’t happen”. The one(s) responsible should, according to teachers and principals, place the topic on the agenda, monitor the implementation, and motivate, inspire and coach teachers.

*Sharing ideas*. Teachers and principals both indicated that no extensive instruction or course for teachers is needed when implementing short forms of PA in the classroom. A single workshop can be helpful to get ideas, but thereafter, team members need to share and exchange ideas, tips and tricks. A part of teachers and principals who had a physical education teacher in their school, suggested that he/she could take on a supporting role herein.

*Communication with parents*. Half of the principals stressed the importance of informing parents when deciding to implement additional PA in school. Principal 7 explained: “You need to communicate properly about why you make the choices you make and what effect they have”.

## 4. Discussion

In this study, we aimed to explore the perceptions of teachers and principals with regard to the implementation of PA interventions aimed at improving cognitive and academic performance, and identify characteristics of feasible school-based PA programmes. Our results add new information to the existing literature that has mainly focused on the perceptions of school staff towards the implementation of PA programmes with the purpose of health promotion. The main findings will be discussed in light of the current evidence on the effects of PA for cognitive and academic performance, and can be used in the development of future school-based PA interventions.

### 4.1. Perceptions of Teachers and Principals

In line with earlier process evaluation studies, teachers and principals in our study had positive attitudes towards PA, attributed to academic, health, and social-emotional benefits for their students [14,15,17,19,29]. Similar to Brown and Elliot [29], our participants particularly mentioned the benefits of PA for learning (i.e., increasing attention), which was a major argument in their expressed willingness to implement additional PA in school. These results are in line with previous studies on health promotion-based programmes suggesting that such programmes need to be complementary to the school curriculum and supportive for learning [12,16]. As the willingness of school staff can affect the success of interventions in schools [15,30], the potential cognitive and academic benefits of PA may facilitate the implementation of school-based PA programmes.

Despite the expressed willingness, our participants emphasized similar barriers as found in previous studies that evaluated the implementation of PA interventions focusing on health outcomes, with a lack of time as the most prominent barrier [14,17,19,22,31]. Therefore, we conclude that the perceptions of our participants with regard to the potential benefits of PA for cognitive and academic performance do not outweigh the perceived time barrier in schools. The accountability of reaching academic targets, that are subject to governmental assessments, seems to determine schools’ priorities and heavily influence teachers’ and principals’ perceptions of the feasibility of school-based PA programmes. Therefore, in order to prioritize and increase the time that can be devoted to PA programmes in schools, future research should gather insight in possibilities to adapt policies at governmental levels [29].

Participants’ feeling of a lack of time due to pressures on academic performance seemed contrary to their expressed willingness to use PA to improve learning, as the latter suggests that PA can help improve academic performance. Our results suggest that teachers and principals perceive PA as a tool that can be used to restore attention in the short-term, rather than a structural intervention that supports them in improving academic performance in terms of students’ grades. The majority of the teachers mentioned that they would implement PA only at times when students needed a break from learning, for example when they lost their focus. This aligns with the literature on the acute effects of PA for cognitive performance, which suggest that PA breaks can provide immediate improvements in for example attention and on-task behaviour [4,32].

None of our participants related PA to students’ grades or referred to scientific evidence when talking about the academic benefits of PA. This could suggest that the current level of evidence does not convince school staff of the long-term effects of structural PA interventions on cognitive and academic performance [20,33], or that they are not aware of the results of scientific research, which is often the case in school practice [34]. Summarizing the most recent reviews, we can conclude that, although small effects of PA on academic performance are reported, there are still many inconsistencies and obscurities in the literature, complicating communication of clear recommendations to policy makers for school practice [20,35,36].

Teachers and principals stressed that evidence on the benefits of PA for cognitive and academic performance is needed to gain team support, which they considered crucial when attempting to implement additional PA in school. Participants furthermore reported that the implementation process needs to be organized within the school, under the supervision of an internal PA coordinator or PA commission, which is in line with earlier studies [12,31]. Despite convincing evidence of the academic benefits of PA, the overwhelming evidence on health benefits may justify integration of theory on the academic as well as health benefits of PA in teacher training programmes.

The perceptions of the teachers and principals in our study were often similar, although there were some differences that align with their different roles. In general, teachers focused mostly on practical issues that they face in their daily routines (e.g., the use of PA as a means to restore children’s attention spans, lack of time, usage frequency), while principals also expanded on organizational barriers such as a lack of financial support and restricted availability of space. Additionally, establishment of policy and communication with parents were important to principals. Earlier studies suggest that principals, besides initiating the implementation, have an essential role in supervising and supporting the implementation process [14,29]. However, the participants in our study recommended to assign the supervision of PA implementation to a “PA coordinator(s)”, which is not necessarily the principal. In The Netherlands it is common that teachers fulfil the role of “coordinator”, as shown by the presence of several “coordinators” in schools (e.g., arithmetic coordinator, information and communications technology (ICT) coordinator, English coordinator). Furthermore, several principals reported the importance of integrating additional PA in the school policy. In line with an earlier study [31], our findings indicate that this is not only the decision of the principal, but it rather needs to be a shared decision of all school staff members.

### 4.2. Feasible School-Based PA Interventions

Due to the perceived time barrier, and financial and space constraints, teachers and principals indicated that additional PA moments in the school-setting are most feasible when implemented in the classroom, carried out by the classroom teacher. In line with earlier studies, teachers and principals suggested that PA programmes need to be short and easy to implement [1,18]. More specifically, they recommended PA programmes that are “ready-to-use”, i.e., providing materials that require little or no preparation time from the teacher. For example: PA breaks presented in a book, from which teachers can choose one to perform with their students, or PA breaks in digital formats, such as movie clips that can be started on the computer. According to teachers and principals PA must be limited to 1–5 min, up to a maximum of 10 min. In this respect, it must be noted that only two studies examined the acute effects of five-minute PA breaks, but found no improvements in cognitive performance (executive functioning and attention) [37,38]. In contrast to studies which used standardized tests to measure cognitive performance, teachers have repeatedly reported to observe improvements in attention after short periods of PA in their classroom [14,15,17,18,19], which was also mentioned by some teachers in the current study. Therefore, it is important that future research includes a measure of teachers’ experiences in addition to standardized performance tasks when evaluating cognitive effects of short PA interventions. This could result in stronger and more practice-based evidence for PA related effects in the school setting.

Although teachers in the study of McMullen et al. [18] preferred PA to be connected to academic content, teachers and principals in our study saw most opportunities in PA breaks during or between academic lessons, followed by PA integrated with academic content. In line with Stylianou et al. [14], this indicates that teachers see PA as a break from learning to restore attention rather than a way to learn. However, it could also indicate that our teachers are not familiar with integrating PA with academic content in higher grades, as supported by their concerns for the appropriateness of this type of PA in grades 5 and 6.

Furthermore, teachers and principals discussed that PA programmes must be fun, varied, challenging and age-appropriate; factors that have been found previously as facilitators of PA engagement in children and adolescents [8,17,39]. As indicated by several of the teachers in this study, we recommend to involve 5th and 6th grade students in the development of PA programmes in order to explore what fun, varied and challenging PA programmes look like according to them.

Lastly, many principals looked for opportunities to implement additional PA outside academic learning hours, for example during recess and after school. This finding is not surprising, considering their role, and again reflect the competing priorities within the school curriculum.

### 4.3. Recommendations for Research and Practice

Looking at the current line of research, we established a gap between the forms of PA that teachers and principals consider most feasible in school (i.e., classroom-based PA, ≤10 min), and the forms of PA that have been examined and proved effective in research studies (i.e., acute PA with a minimum duration of 10 min [32], and chronic PA interventions with durations of 15 min or longer per session [20]). Therefore, we recommend future research to investigate the cognitive and academic effects of forms of PA programmes that are, according to teachers and principals, feasible in school. In particular, studies should focus on very short PA breaks in the classroom (≤5 min), and examine the acute as well as long-term effects of repeated use of short PA breaks in the classroom. Our findings also confirm the importance of involving school staff in the development of school-based PA interventions, in order to develop interventions that are more likely to be implemented successfully in daily school practice [8]. Furthermore, we recommend research on health promotion-based PA interventions to include additional measures of cognitive and academic performance, as we revealed that these outcomes are important for teachers’ and principals’ willingness to implement PA programmes in school. In addition, this would help to form a better picture of the evidence base of PA for cognitive and academic performance, which is needed to inform policy makers and school professionals, hence increasing the chance of successful PA implementation in schools.

Our findings have also implications for school practice. As the perceived lack of time is a major issue in schools, this needs to be taken into account when aiming to implement additional PA in school. Therefore, we recommend principals and teachers to look for opportunities to implement PA in the classroom, since this requires relatively little time, finances and space (e.g., there is no need for hiring additional staff and/or PA facilities, like a sports hall). We also suggest schools to discuss strategies to lower the perceived time barrier, and look for ways to prioritize the use of PA in school. Herein, the potential benefits of PA for learning could be central. Decisions should be made by both principal and teachers, as team support seems essential for successful and sustainable implementation. We recommend enthusiastic teachers to take a leading role in the promotion of PA in school. Principals can support these teachers and encourage them to take a role as “PA coordinator”. We also recommend principals to provide/organize a workshop on the background and practical use of PA in the classroom. In particular, we suggest to discuss the cognitive and academic benefits of PA perceived by teachers, as well as the current scientific evidence. In this respect, it is important to note that although the scientific evidence needs to be strengthened, we can safely conclude that additional time spent on PA will not negatively influence cognitive and academic performance [20]. This may stimulate teachers to spend more time on implementing PA in their classroom. Lastly, we recommend teachers to exchange ideas and experiences with each other, and set up regular follow-up meetings, which might promote PA as part of their daily school routines.

### 4.4. Strengths and Limitations

A strength of our study was that data saturation was achieved, and that we consulted participants from several regions across The Netherlands. Other strengths were the inclusion of the audit trail and the interview guides, which provided transparency and ensured trustworthiness of our study, thereby enabling replication. Also, we provided new information that could be used by researchers in future studies on school-based PA interventions, as well as ideas and suggestions that could be used by schools that are looking for ways to implement additional PA in school. A limitation was that we faced a group of fairly motivated teachers and principals, despite the attempt to recruit and include teachers and principals that were also sceptical on the topic. This was supported by the response rate of 26%, and limited the generalisability of the results to less motivated teachers and principals. However, even in this group of participants, several barriers were apparent that would also be applicable to less motivated school staff. Moreover, our findings may not be generalisable to teachers and principals in other countries with different school systems and cultures. Another limitation was that due to time constraints and short availability of interviewers, we could not start data analysis during the data collection period. Therefore, we were not able to adjust questions during the course of the study, which could have provided us with more in-depth information into some aspects of the topic.

## 5. Conclusions

In summary, the 5th and 6th grade teachers and primary school principals who participated in the current study had positive attitudes towards PA. They expressed their willingness to implement additional PA in school, in which the academic benefits was an important argument. However, teachers and principals highlighted similar barriers as found in studies on implementation of health promotion-based PA interventions, with time constraints as the most important barrier. Our participants emphasized that feasible PA programmes should be short (i.e., few minutes), provided in “ready-to-use” materials, and be executed in the classroom by the classroom teacher. According to our participants, PA could be best used as break at moments that attention spans decline. Future research on the effects of such PA programmes on cognitive and academic performance is needed to provide policy makers and school staff with clear information on its benefits for school practice. Furthermore, we recommend schools to look for opportunities to implement PA in the classroom, and discuss strategies to lower time barriers and prioritize PA in school.

## Appendix A

**Table A1:** Interview guides.

Subgroup	Interview Questions
Teachers	**Initial Question**
	Do you include some form of physical activity during your lessons? If so, what are your experiences? If not, can you say why not?
	**Core Questions**
	1. What do you think of the idea of introducing additional physical activity at school to improve cognitive performance and academic performance? 2a. What possibilities do you see in general for additional physical activity at school? 2b. If so, with what goal would you introduce additional physical activity? Does it make any difference to you whether this is introduced to improve health or to improve cognitive skills or academic performance? 3. What possibilities can you see for introducing physical activity during or in between your lessons in the classroom? 4. What, in your opinion, is the best way to provide additional physical activity at school? 5. What, in your opinion, is required to make a success of introducing additional physical activity in the school setting? And what is required to make this a permanent component of the curriculum? 6. What, in your opinion, are the most important points to consider when implementing additional physical activity in the school setting?
Principals	**Initial Question**
	What is the vision and policy of the school with respect to physical activity? In what way is attention focused on physical activity in the school curriculum? Is some form of physical activity implemented by the teachers in their lessons?
	**Core Questions**
	1. What do you think of the idea of introducing additional physical activity at school to improve cognitive performance and academic performance? 2a. In your opinion, what possibilities are there for introducing additional physical activity in the school curriculum? 2b. If so, with what goal would you introduce additional physical activity? Does it make any difference to you whether this is introduced to improve health or to improve cognitive skills or academic performance? 3. In your opinion, what is the best way to embed additional physical activity in the school curriculum? How do you envisage this in practice? 4. In your opinion, who is the most suitable person to provide additional periods of physical activity? 5. What, in your opinion, is required to make a success of introducing additional physical activity in the school setting? And what is required to make this a permanent component of the curriculum? 6. What, in your opinion, are the most important points to consider when implementing additional physical activity in the school setting?
